# Electrochemical Analysis and Inhibition Assay of Immune-Modulating Enzyme, Indoleamine 2,3-Dioxygenase

**DOI:** 10.3390/ph18030352

**Published:** 2025-02-28

**Authors:** Yasuhiro Mie, Chitose Mikami, Yoshiaki Yasutake, Yuki Shigemura, Taku Yamashita, Hirofumi Tsujino

**Affiliations:** 1 Bioproduction Research Institute, National Institute of Advanced Industrial Science and Technology (AIST), Sapporo 062-8517, Japany-yasutake@aist.go.jp (Y.Y.); 2 Computational Bio Big-Data Open Innovation Laboratory (CBBD-OIL), AIST, Tokyo 169-8555, Japan; 3 Graduate School of Pharmaceutical Sciences, Osaka University, Osaka 565-0871, Japanhtsujino@phs.osaka-u.ac.jp (H.T.); 4 School of Pharmacy and Pharmaceutical Sciences, Mukogawa Women’s University, Nishinomiya 663-8179, Japan; 5 Museum Links, Osaka University, Osaka 560-0043, Japan

**Keywords:** immunosuppressive enzyme, inhibition screening, electrochemical assay, redox control, nanoporous electrode

## Abstract

**Background:** An accurate and rapid analysis of human indoleamine 2,3-dioxygenase (hIDO) is crucial for the development of anticancer pharmaceuticals because of the role of hIDO in promoting tumoral immune escape. However, the conventional assay of hIDO is limited by interference from reductants, which are used to reduce the heme iron to begin the hIDO catalytic reaction. **Methods:** A direct electrochemical method was applied to drive the hIDO reaction. **Results:** The nanostructured gold electrode enabled the electrochemical reduction of the heme iron of hIDO1. In the presence of substrates (tryptophan and oxygen), a bioelectrocatalytic current was observed, confirming an electrochemically driven hIDO reaction. A well-known inhibitor of hIDO, epacadostat, hindered this catalytic signal according to its concentration, demonstrating the rapid evaluation of its inhibition activity for the hIDO reaction. Through an in silico study using the proposed electrochemical assay system, we discovered a strong inhibitor candidate with a half-maximal inhibitory concentration of 10 nM. **Conclusions:** An accurate and rapid assay system in drug discovery for hIDO and kynureine pathway-targeted immunotherapy has been developed.

## 1. Introduction

The extremely complex process of clearing foreign and aberrant cells while preventing autoimmunity is performed to maintain multicellular life. This finely tuned balance is frequently mediated by enzymes involved in central metabolism [1]. One such enzyme is human indoleamine 2,3-dioxygenase 1 (hIDO1). It catalyzes the oxidative cleavage of L-tryptophan (Trp) to *N*-formylkynurenine (NFK) by opening the five-membered ring of Trp using molecular oxygen (O_2_) [2,3,4]. Most of Trp is metabolized through the kynurenine (KYN) pathway, and hIDO1 acts as the first and rate-limiting step of the pathway. Thus, this enzyme plays an important role in controlling the relative TRP flux. The overactivation of hIDO1 leads to the upregulation of the KYN pathway, contributing to anxiety, psychosis, cognitive decline, and neurodegenerative disorders [5,6,7,8]. It has been recently discovered that cells that express hIDO1 activity can profoundly alter their environment to suppress the immune response by depleting Trp (the key nutrient required for T-cell activation) and by promoting the production of immunosuppressive kynurenine metabolites [9,10]. Consequently, hIDO1 has attracted attention as an important therapeutic drug target [11]. In addition, a second isoform of hIDO, hIDO2, was discovered and observed to be dysregulated in cancer cells [12], where it may play a crucial role in suppressing antitumor immunity similar to hIDO1.

Considering that hIDO functions as an immunosuppressor, the strong inhibition of the enzyme is expected to deteriorate cancer cell growth through antitumor immunity. Thus, inhibitor screening is essential for drug discovery. To promote this, the accurate and rapid analysis of hIDO is important. hIDO contains heme iron as the active site, which should be reduced to initiate the Trp oxidation reaction. To date, ascorbate and methylene blue (MB) have commonly been used to determine hIDO activity, where ascorbate reduces the MB, which functions as an electron mediator to reduce the heme iron [13,14,15]. However, this system presents certain challenges. Sono et al. indicated that such a reducing system reduced ferric hIDO by only 25–40% under certain conditions [16]. Such an incomplete reduction of hIDO was supported by resonance Raman spectroscopy [17] and attributed to the relatively low redox potential (*E*^0^′) of the hIDO heme iron. Furthermore, the MB reduced by ascorbate was reoxidized by dioxygen (a substrate for the Trp oxidation reaction by hIDO) [18]. This suggested a difficulty in controlling the oxygen concentration during the assay [16]. For hIDO2, Yuasa et al. reported that the ascorbate and MB system significantly affected the enzymatic reaction [19]. They demonstrated that ascorbate is a weak competitive inhibitor, and that MB inhibits hIDO2 at low Trp concentrations while enhancing the activity at higher Trp concentrations. Owing to such phenomena, a more simple and useful assay system for hIDOs is urgently required to develop immunotherapeutic drugs.

The electrochemical method is a useful technique for controlling the electron transfer reaction (oxidation/reduction state) of redox molecules, using an electrode as an electron donor or acceptor by applying the electrochemical potential. Direct electron transfer between the electrode and redox enzyme active site (e.g., heme iron in IDO) is often difficult to achieve because the active site is generally located in polypeptide chains. Thus, the enzyme molecule at the electrode surface must be properly oriented to minimize the distance of electron transfer since the rate of electron transfer exponentially decreases with an increase in distance [20,21,22]. Recently, conductive nanomaterials, such as nanocarbons and metal nanoparticles, have been frequently used to modify the surface of electrode substrates. Furthermore, the given nanostructured surface is effective for achieving direct electron transfer with the enzyme active site [23,24,25]. In addition, the nanostructurization of the electrode materials is also useful. Nanoporous gold (NPG) prepared via the anodization of a planar gold surface is a prospective material. This is because of its advantages of high conductivity, chemical inertness, physical stability, reusability, high catalytic activity, and facile surface modification [26,27]. In addition, the anodization strategy for producing NPG is extremely simple, facile, and rapid. We recently developed a strategy for controlling the NPG surface structure [28] and immobilized heme redox enzymes, such as cytochrome P450, neuroglobin, and cytoglobin, on structure-controlled NPG. Thereafter, we observed that the NPG structure strongly enhanced the electron transfer reaction with the electrode and heme iron of these enzymes [29,30,31]. To the best of our knowledge, there have been no reports on the electrochemical analysis of hIDO with direct electron transfer at an electrode.

Here, we examined the redox state control of hIDO1 using an NPG electrode with direct electron transfer and demonstrated the electrochemical analysis of hIDO1 without interference from chemical reductants. An efficient electrochemical inhibitor assay system was constructed, and a strong inhibitor candidate was discovered using the system.

## 2. Results and Discussion

### 2.1. Electrochemical Redox Control of hIDO1 Using NPG Electrode with Direct Electron Transfer

**Figure 1:**
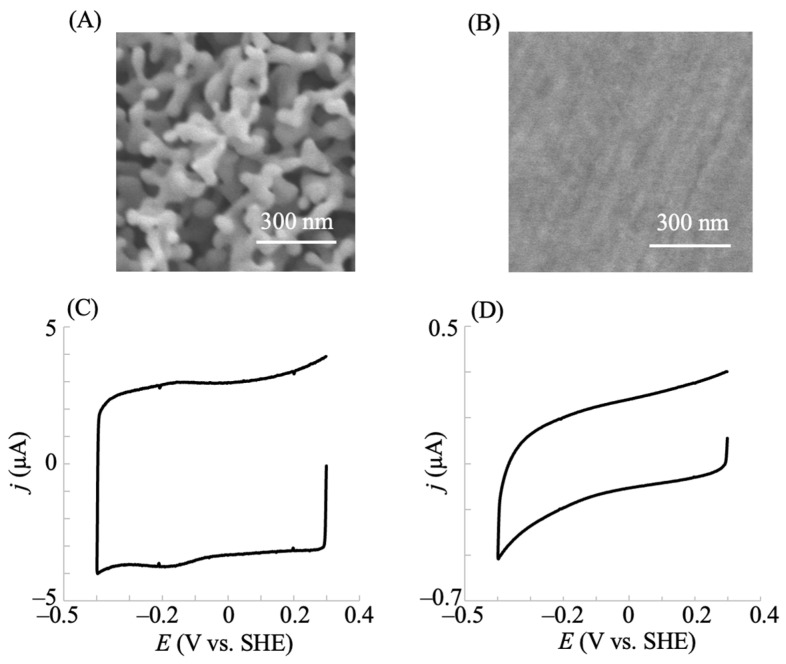
SEM images of the NPG (**A**) and planar gold (**B**) electrodes within the range of 1 × 1 µm, and cyclic voltammograms of IDO1 immobilized on the NPG (**C**) and planar gold (**D**) electrodes in a 0.1 M phosphate buffer solution containing 0.1 M NaCl at a scan rate of 0.2 V s^−1^.

The direct electron transfer reaction between the enzyme active site and the electrode surface provides a simple system for the electrochemical investigation of the enzyme. Thus, we investigated the electrode surface structure to achieve direct electron transfer. The voltammetric responses of hIDO1 immobilized on the planar gold and NPG electrodes obtained in a phosphate buffer solution (pH = 7.5) containing 0.1 M NaCl at a potential scan rate of 0.2 V s^−1^ are shown in Figure 1. Figure 1A,B show the morphology of the surfaces of the NPG and planar electrodes, respectively, with the nanostructure comprising pores and ligaments on the NPG surface. The immobilization was conducted at the hydroxy-terminated thiol-modified gold surface through covalent bonding with the hydroxy group of the hIDO1 surface. As shown, a clear couple of reductive and oxidative currents were observed at the NPG electrode of hIDO1 (Figure 1C), indicating rapid direct electron transfer between the heme iron of hIDO1 and the electrode surface. The *E*^0^′ value obtained from the voltammogram was −145 mV at pH 7.5. This value was comparable to that (–68 mV) obtained at a pH of 7.0 measured using the dye(s) titration method [32], considering the difference in the measured method and pH condition for the one-electron transfer reaction (Fe^3+^ + e^−^ = Fe^2+^). The charges of the oxidative and reductive current signals corresponded to 1.0 ± 0.25 nmol cm^−2^, indicating the monolayer coverage of hIDO1. The intensity of the faradaic (oxidative and reductive) currents from hIDO1 practically remained unchanged within the tested period of up to 24 h. The results showed the electrochemical control of the redox states of the heme iron in hIDO1. Conversely, the planar gold electrode did not exhibit a faradaic current signal, suggesting the usability of the proposed NPG electrode for electrochemically investigating hIDO1. The direct electron transfer could have resulted from the curvature effect of the nanoporous structure [22].

**Figure 2:**
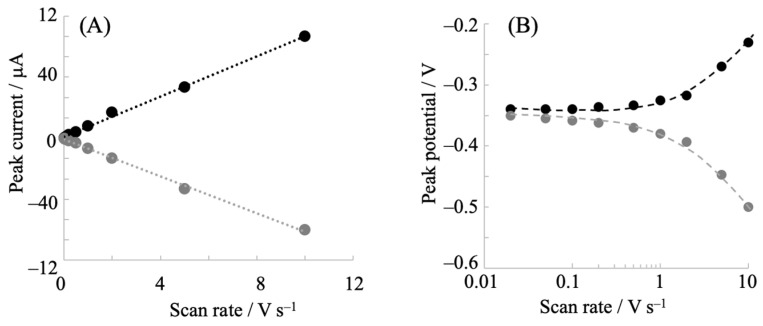
Relationship between (**A**) the oxidative (black circle) and reductive (gray circle) peak currents and (**B**) the oxidative (black circle) and reductive (gray circle) peak potentials against potential scan rates for the hIDO1-immobilized NPG electrode. Voltammetry was conducted in a 0.1 M phosphate buffer solution (pH 7.5) containing 0.1 M NaCl under an Ar atmosphere.

**Figure 3:**
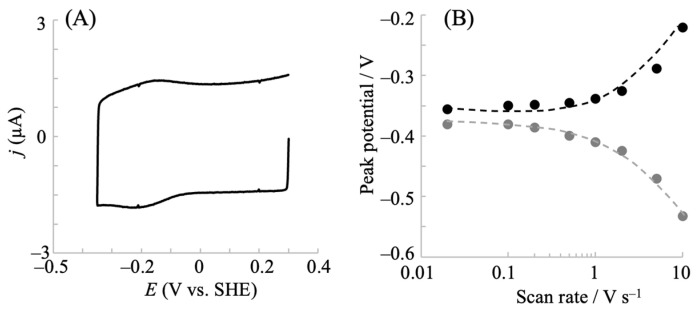
(**A**) Cyclic voltammogram of hIDO1 R231Q mutant immobilized on an NPG electrode in a 0.1 M phosphate buffer solution containing 0.1 M NaCl at a scan rate of 0.2 V s^−1^. (**B**) The corresponding trumpet (peak potential vs. potential scan rate) plot for R231Q. The gray and black circles show reductive and oxidative potentials, respectively.

The cyclic voltammograms of hIDO1 were analyzed to understand its electrochemical properties. Figure 2 shows the potential scan rate dependence of the voltammetric behavior. The oxidative and reductive peak currents exhibited a linear relationship with the scan rate (Figure 2A), indicating that the electrochemical responses originated from the surface-bound hIDO1. The peak separation between the anodic and cathodic peak potentials increased with the potential scan rate. This provided an apparent heterogeneous electron transfer rate constant (*k*_s_′) of 21 s^−1^ from the fitting of a trumpet plot (peak position vs. scan rate, Figure 2B), using a simulation program developed by Jeuken et al. [33]. The value of the constant was comparable to that of neuroglobin, which exhibited similar heme iron coordination structures during the redox reaction, immobilized on an NPG electrode (46 s^−1^) [30]. We additionally investigated the mutated hIDO1, R231Q, whose activity for Trp cleavage was considerably low (described below). The mutant showed a couple of redox peak currents (Figure 3) similar to those of the wild type (WT), indicating rapid electron transfer to the electrode surface. Although the *E*^0^′ value was slightly (20 mV) more negative, the *k*_s_′ of the R231Q mutant estimated from the trumpet plot was 17 s^−1^, which was close to that of the WT. This indicated that this mutation had no significant influence on the electron transfer kinetics, and that the structural changes that occurred during the redox reaction of hIDO1 for both the WT and R231Q were similar.

### 2.2. Electrocatalytic Reductive Reaction with hIDO in the Presence of Molecular Oxygen and Trp

**Figure 4:**
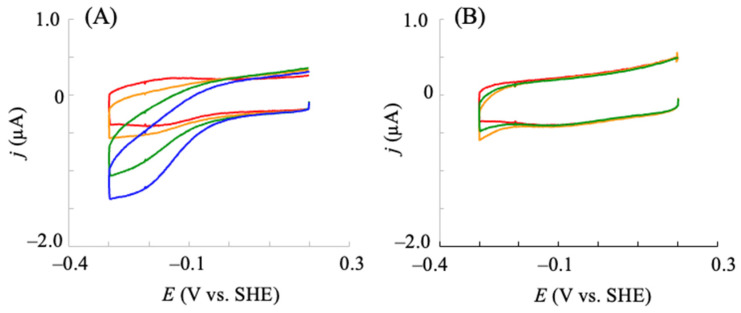
Cyclic voltammograms of NPG electrodes modified with hIDO1 (**A**) and bovine serum albumin (**B**) in a phosphate buffer (pH = 7.5) containing 100 mM NaCl upon the addition of molecular oxygen at 20 mV s^−1^. [O_2_]: 0 (red line), 11 (orange line), 20 (green line), and 38 (blue line) μM in (**A**), and 0 (red line), 35 (orange line), and 48 (green line) μM in (**B**).

Considering that the electrochemical redox control of hIDO1 was successfully achieved using NPG, its electrocatalytic reaction was investigated in the presence of substrates. First, we investigated the reaction in the presence of molecular oxygen (O_2_, one of the substrates of hIDO1). As shown in Figure 4A, an electrocatalytic reductive current was observed in the presence of O_2_, which appeared at the *E*^0^′ in the non-turnover voltammogram of hIDO1 in the absence of O_2_ (red line in Figure 4A) at a scan rate of 20 mV s^−1^. The catalytic current increased with an increase in the concentration of dissolved O_2_. Furthermore, the ratio of the reductive currents in the presence of O_2_ compared with that in the absence of O_2_ increased with a decrease in the potential scan rate. The control experiments with NPG electrodes modified with 6-hydroxy-1-hexanethiol (HHT) and HHT coupled with bovine serum albumin did not result in such catalytic currents (Figure 4B). The results showed that the reduced hIDO1 reacted with molecular oxygen. This kind of bioelectrocatalytic response with oxygen has been reported for an electrode immobilized with myoglobin, which has a His residue coordinated to the heme iron at the distal site, similar to hIDO1. The reaction was recognized as the electrochemical catalytic reduction of O_2_ to produce hydrogen peroxide [34,35,36]. The oxidation reaction of the ferrous heme iron by the produced hydrogen peroxide to ferric iron was suggested from the voltammograms [37]. hIDO1 has a heme iron coordination structure similar to that of myoglobin. Thus, we expect that the bioelectrocatalytic reaction for molecular oxygen occurred at the hIDO1-immobilized NPG electrode, similar to the myoglobin case, as follows:

hIDO1(Fe^3+^) + e^−^ ⇌ hIDO1(Fe^2+^) (1)

hIDO1(Fe^2+^) + O_2_ → hIDO1(Fe^2+^)–O_2_ (2)

hIDO1(Fe^2+^)–O_2_ + 2H^+^ + 2e^−^ → hIDO1(Fe^2+^) + H_2_O_2_ (3)

2hIDO1(Fe^2+^) + H_2_O_2_ + 2H^+^ → 2hIDO1(Fe^3+^) + 2H_2_O (4)

The electrochemically reduced hIDO1 (Equation (1)) was bound to oxygen to become an oxy form (Equation (2)), and it was converted into the deoxy form with the production of hydrogen peroxide at the electrode (Equation (3)). Thereafter, the deoxy form was oxidized to the ferric form by the hydrogen peroxide (Equation (4)).

**Figure 5:**
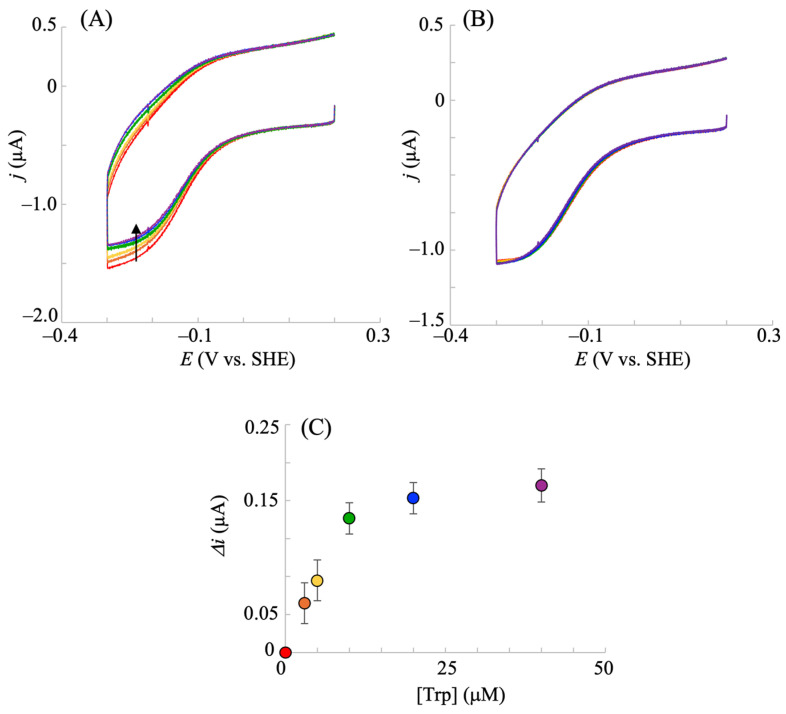
Cyclic voltammograms of hIDO1 WT-immobilized (**A**) and R231Q-immobilized (**B**) NPG electrodes upon the addition of Trp in a phosphate buffer (pH = 7.5) containing 100 mM NaCl and ca. 40 μM molecular oxygen. The Trp concentration was increased from 0 to 40 μM (red: 0; orange: 2.5; yellow: 5; green: 10; blue: 20; purple: 40 μM). The corresponding plot for [Trp] vs. the decreased current (**C**). Values are presented as the mean ± SD (*n* = 3 independent experiments). The arrow in (A) indicates the decrease of the catalytic current upon addition of Trp.

Next, electrocatalysis using the hIDO1-immobilized electrode in the presence of Trp and oxygen was investigated. Prior to the addition of Trp to the system, voltammetry was conducted in the presence of 35–45 µM oxygen, and the aforementioned hIDO1-based oxygen reductive catalysis was confirmed. This range of oxygen concentrations was selected because it includes physiologically relevant levels [38]. The addition of Trp lowered the electrocatalytic oxygen reduction current according to its concentration (Figure 5A). The electrochemically reduced hIDO1 (hIDO1(Fe^2+^)) was first bound to oxygen to become the oxy form (hIDO1(Fe^2+^)–O_2_) (Equations (1) and (2)). Thereafter, Trp was bound to the distal heme pocket of hIDO1(Fe^2+^)–O_2_ to produce a ternary complex, hIDO1(Fe^2+^)–O_2_–Trp (Equation (5)).

hIDO1(Fe^2+^)–O_2_ + Trp → hIDO1(Fe^2+^)–O_2_–Trp (5)

hIDO1(Fe^2+^)–O_2_–Trp → IDO1(Fe^2+^) + KYN (6)

The ternary complex converted Trp into NFK. The *k*_cat_ range for the NFK formation by hIDO1 within an oxygen concentration range of 30–50 µM was reported to be 7.1–8.5 s^−1^ [38]. Thus, upon the addition of Trp, we expected the reaction of Equation (3) in the electrocatalytic oxygen reduction by hIDO1 to be hindered by the consumption of hIDO1(Fe^2+^)–O_2_ species by the inherent Trp cleavage reaction of hIDO1 (Equations (5) and (6)). Therefore, the decrease in the bioelectrocatalytic oxygen reductive signal upon the addition of Trp (Figure 5A) was reasonable, indicating that this decrease can be a measure of hIDO1 Trp cleavage catalysis.

To further confirm the electrochemical assay of Trp catalysis by hIDO1, we conducted measurements with R231Q-mutated hIDO1, which exhibited lower catalytic activity for Trp conversion compared with that of the WT. The *k*_cat_/*K*m of R231Q was smaller by three orders of magnitude than that of the WT. As shown in Figure 5B (red line), the R231Q mutant exhibited an electrocatalytic reductive current in the presence of oxygen and the absence of Trp, similar to the WT, suggesting that the aforementioned oxygen reduction reaction occurred. However, no significant change was observed with the addition of Trp (Figure 5B), which is different from the WT case (Figure 5A). R231Q has considerably less binding of Trp by the mutation at the position which connects to Trp, while oxygen can be bound to its heme iron. Thus, the above electrochemical observation for the WT and R231Q is consistent with these facts. The Trp concentration that reduced the current by half for the WT was estimated to be 7 µM (Figure 5C). This was slightly smaller than the reported *K*_M_ obtained via the conventional method for converting Trp into NFK (18–25 µM) [38,39]. The addition of MB in the conventional strategy has been reported to decrease the affinity of human IDO1 for Trp [40]. Therefore, the value obtained through the present method could be reasonable because MB was absent in the electrochemical system. The results showed that the hIDO1 activity can be electrochemically evaluated using the present NPG electrode without the conventional reducing agents that affect hIDO reactions.

### 2.3. Electrochemical hIDO Inhibition Assay

**Figure 6:**
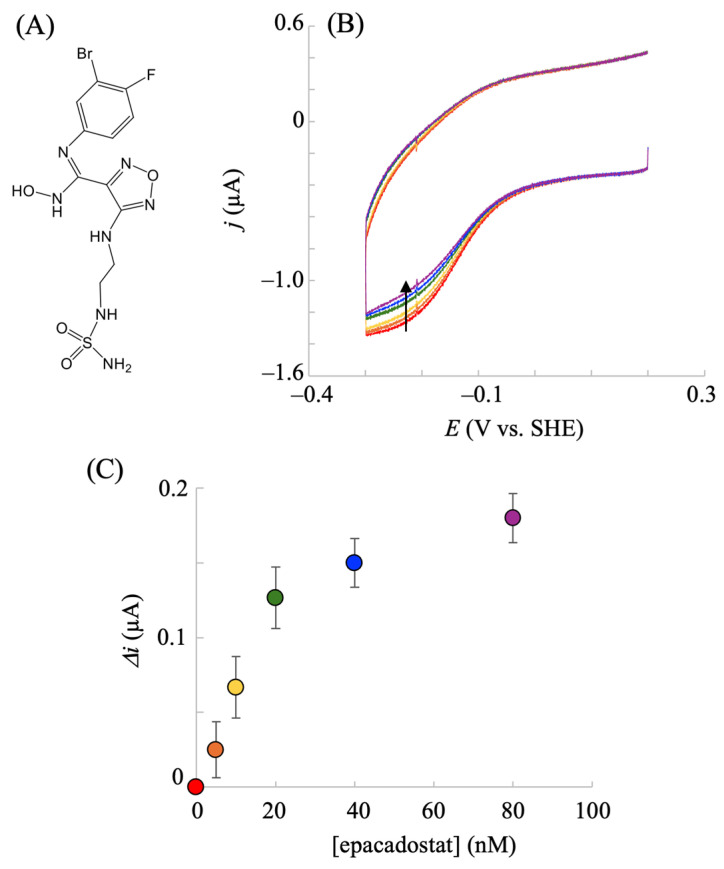
Chemical structure of epacadostat (**A**). Voltammetric responses of the hIDO1-modified NPG electrode upon the addition of epacadostat (red: 0; orange: 5; yellow: 10; green: 20; blue: 40; purple: 80 nM) in a phosphate buffer (pH = 7.5) containing 100 mM NaCl, 40 μM O_2_, and 500 μM Trp at a scan rate of 0.02 V s^−1^ (**B**). The corresponding plot for [epacadostat] vs. the decreased current (**C**). Values are presented as the mean ± SD (*n* = 3 independent experiments). The arrow in (**B**) indicates the decrease of the catalytic current upon the addition of epacadostat.

The inhibition of hIDO1 for converting Trp into NFK is important for developing anticancer therapeutics [11]. As described above, the electrochemically driven hIDO1 reaction was achieved, and rapid analysis of the inhibition reaction was expected using the electrochemical strategy. Thus, we investigated whether a change in the electrochemical response of hIDO1 was observed after the addition of the inhibitor(s). First, the well-known hIDO1 inhibitor epacadostat (Figure 6A) was examined. Upon the addition of epacadostat, the stable electrocatalytic response of hIDO1 in the presence of O_2_ and Trp decreased (Figure 6B). The degree of the decrease increased with an increase in the concentration of the inhibitor added to the electrochemical system and practically plateaued above a certain concentration. The apparent half-maximal inhibitory concentration (IC_50_) was estimated to be 15 nM from the plot of the inhibitor concentration vs. the degree of decrease in the catalytic current (Figure 6C). This electrochemically calculated value is comparable to reported values (7–15 nM) obtained via cell-based assays [41]. A biochemical assay for isolated/purified hIDO1 using ascorbate and MB provided IC_50_ values above those obtained via cell-based assays [41,42]. The authors claim that the phenomenon results from the complexity of the ascorbate and MB system. Therefore, the results show that the present electrochemical system with the hIDO1-immobilized NPG electrode is suitable for the inhibition screening of this immune-modulating enzyme without the influence of the aforementioned additives.

**Figure 7:**
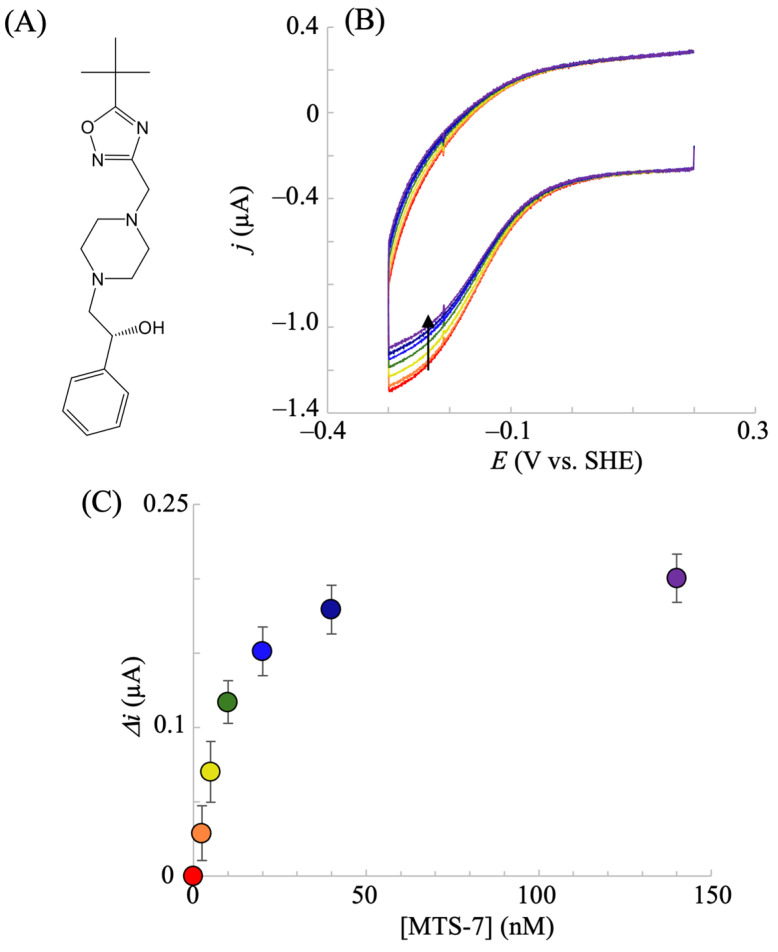
Chemical structure of the predicted candidate (MTS-7) by MTS calculation (**A**). Voltammetric responses of the hIDO1-modified NPG electrode upon the addition of MTS-7 (red: 0; orange: 2.5; yellow: 5; green: 10; blue: 20; navy: 40; purple: 140 nM) in a phosphate buffer (pH = 7.5) containing 100 mM NaCl, 40 μM O_2_, and 500 μM Trp at a scan rate of 0.02 V s^−1^ (**B**). The corresponding plot for [epacadostat] vs. the decreased current (**C**). Values are presented as the mean ± SD (*n* = 3 independent experiments). The arrow in (**B**) indicates the decrease of the catalytic current upon the addition of MTS-7.

**Figure 8:**
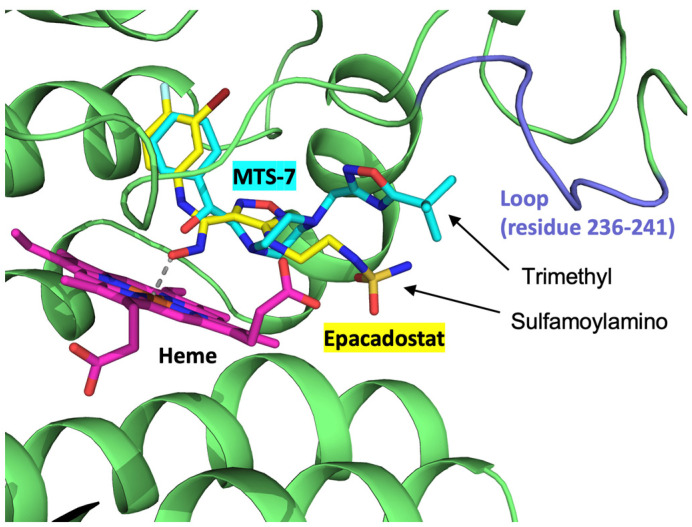
The best docking pose between hIDO1 (PDB code, 5WN8) and MTS-7. The bound epacadostat observed in the structure of 5WN8 is shown. The carbon atoms of MTS-7, epacadostat, and heme are shown in cyan, yellow, and magenta, respectively. The hIDO1 structure is shown in green ribbon representation. The loop region of hIDO1 in the vicinity of the trimethyl of MTS-7 (residues 236–241) is colored light blue.

Next, we investigated whether the present electrochemical inhibition assay could be used to identify new IDO inhibitors. Ten compounds with top hits in in silico screening were purchased and examined via electrochemical hIDO1 analysis in the presence of O_2_ (40 μM) and Trp (500 μM). Most of the tested compounds did not affect the electrochemical responses when added to the system. One compound, whose chemical structure is shown in Figure 7A, selected by the multiple target screening (MTS) method (MTS-7), considerably decreased the electrochemical reductive signal (Figure 7B). In addition, this compound did not significantly alter the electrochemical response of neuroglobin (heme protein irrelevant to Trp metabolism), suggesting that MTS-7 was not a promiscuous inhibitor for the electrochemical system. This indicates the inhibition of the hIDO1 catalytic reaction of Trp metabolism by MTS-7. It is noted that the commercially available MTS-7 is a racemic mixture. The plot of the candidate concentration and the decrease in the reductive current (enzymatic activity) shown in Figure 7C provides an apparent IC_50_ value of approximately 10 nM. This value is comparable with that of epacadostat and indicates the potency of this strong inhibitor for the hIDO1 reaction. Molecular docking simulations were performed to assess the binding compatibility of MTS-7 to the active site pocket of hIDO1. The best docking pose (binding affinity, −8.3 kcal/mol) was compared with the epacadostat model bound to the hIDO1 active site determined via a crystallographic study (PDB code, 5WN8) [2]. The top 10 docking poses are summarized in Supplementary Table S1. Except for docking poses #4 and #7, the remaining eight poses are well superimposed within the active site, showing similar binding conformations. The best pose between hIDO1 and MTS-7 is drawn in Figure 8, superimposed on the bound epacadostat model. As shown, the phenyl group of MTS-7 practically occupied the same location in the active site pocket of hIDO1 as the 3-bromo-4-fluorophenyl moiety of epacadostat. Contrarily, the hydrophobic trimethyl group of MTS-7 lay adjacent to a loop structure comprising amino acid residues 236–241 of IDO1. This position differs from that occupied by the solvent-exposed hydrophilic sulfamoylamino moiety of epacadostat. Overall, MTS-7 appears to be able to fit well and bind to the active site pocket of hIDO1 by isolating the hydrophobic trimethyl group from the solvent.

Finally, we performed the conventional hIDO1 inhibition assay for MTS-7 with ascorbate and MB as an electron supply system. In contrast to the result obtained via the aforementioned electrochemical assay, strong inhibition was not observed. We suspect that the considerably higher (three orders of magnitude) concentration of MB inhibited the binding for MTS-7. Although the sulfamoylamino group of epacadostat was exposed to the solvent region, the trimethyl groups of MTS-7 lay alongside amino acids 236–241 in the heme pocket, where the hydrophobic MB may be located, to transfer electrons to the heme iron. The proposed method is free from this kind of concern because reductants and/or electron transfer mediators are not required.

Thus, we conclude that the present electrochemical system with an NPG surface is an adequate analytical method for hIDO1 activity without interference from reductants and electron transfer mediators, whose phenomena are observed using the conventional method. The present method is also rapid because of the real-time monitoring of the hIDO1 catalytic reaction, and the calculated Z’-factor for the inhibition assay was 0.59. Hence, we believe that this method can be suitable for high-throughput systems as a primary screening assay.

## 3. Materials and Methods

### 3.1. Reagents

6-Hydroxy-1-hexanethiol (HHT) for the gold surface modification was purchased from Sigma-Aldrich (St. Louis, MO, USA) and was used as received. Epacadostat and inhibitor candidates were obtained from MedChemexpress Co., Ltd (Monmouth Junction, NJ, USA), and Namiki Shoji, Co., Ltd. (Tokyo, Japan), respectively. All the aqueous solutions were prepared using ultrapure water (18 MΩ cm).

### 3.2. Preparation of hIDO1 Enzymes

Following a previous study [43], full-length hIDO1 was expressed in an *Escherichia coli* system, followed by extraction and chromatography to obtain a highly purified enzyme. The gene encoding hIDO1 was ligated into an expression vector (pET3a), and transfected *E. coli* was cultured in a lysogeny broth medium with 0.1 g/L ampicillin (110 rpm, 37 °C). When the culture attained an optical density of 0.6 at 600 nm, isopropyl β-(d) thiogalactopyranoside and hemin were successively added to final concentrations of 5 and 7 µM, respectively, to induce protein expression. Culturing was continued at 4500 *g* and 4 °C for 10 h. Cells were harvested and frozen at −80 °C and lysed in a 20 mM potassium phosphate buffer (pH = 6.5). The soluble fraction was isolated by ultracentrifugation (20,000× *g*), and the supernatant was applied to a hydroxyapatite gel column (BIO-RAD, Hercules, CA, USA), followed by purification using a CM Sepharose column (GE Healthcare, Chicago, IL, USA).

### 3.3. Fabrication of Nanostructured Electrode and hIDO1 Immobilization

A gold disk electrode (Bioanalytical Systems, Tokyo, Japan, ∅ = 3 mm) was polished using diamond and alumina slurries, and its surface was electrochemically cleaned using 0.5 M H_2_SO_4_ serving as a conventional (planar) gold electrode [28]. The cleaned planar electrode was placed into a solution of 0.5 M HCl, and an anodized potential of about ~1.36 V vs. Ag|AgCl|sat.KCl was applied for ~3 min to fabricate the nanoporous gold (NPG) structure, as has been reported [30]. The surface area of the anodized (nanostructured) gold surface was estimated using a voltammogram recorded at a scan rate of 0.1 V^−1^ in H_2_SO_4_ [44], and the surface roughness (*R*f) was calculated by dividing the surface area by the geometrical area. An *R*f below 5 was used in the present study to minimize the effect of the mass transfer limitation of the substrates/inhibitor into the deep pores of the NPG [30]. The morphology of the NPG surface was mainly analyzed by scanning electron microscopy (SEM) characterization (S-4300 FE-SEM, Hitachi Ltd., Tokyo, Japan). The electrode surface was modified with thiol compounds using the 0.5 mM ethanolic solution of each modifier. The hIDO1 molecule was immobilized onto the HHT-coated surface by casting a 0.1 mM hIDO1 solution with 10 mM 1,2-bis (trimethoxysilyl)ethane [45] through a hydroxy group (NPG–O–Ngb).

### 3.4. Electrochemical Measurements and hIDO1 Assay

Voltammetry was conducted utilizing an electrochemical analyzer (CH Instruments Inc., Austin, TX, USA) with a normal three-electrode configuration consisting of a Ag|AgCl|sat.KCl reference electrode, Pt auxiliary electrode, and hIDO1-immobilized working electrode [23]. The potentials reported in this work were converted to the standard hydrogen electrode (SHE). Bioelectrocatalytic measurements were performed by controlling the O_2_ concentration using a mixed gas controller (Yamato Sangyo, Osaka, Japan) [38].

### 3.5. In Silico Study

In silico screening was performed using the program MF myPresto (ver. 3.2, FiatLux, Tokyo, Japan). The multiple target screening (MTS) [46] and docking score index (DSI) [47] methods were applied for the in silico screening against a library consisting of approximately 5,000,000 chemical compounds. An atomic model of the hIDO1 structure for the MTS method was obtained from the Protein Data Bank with accession code 5WN8 [2]. Epocadostat was used as a known active compound (inhibitor) for hIDO1 in the DSI method. Ten commercially available compounds that hit the top rankings (Namiki Shoji Co., Ltd., Tokyo, Japan) were subjected to electrochemical inhibition assays. Molecular docking between hIDO1 and 2-(4-{[5-(2-methyl-2-propanyl)-1,2,4-oxadiazol-3-yl]methyl}-1-piperazinyl)-1-phenylethanol (MTS-7) was performed using the program AutoDock Vina (ver. 1.1.2) [48]. The configured input files were generated using AutoDock Tools (ver. 1.5.6) [49]. The simulation box for docking calculations that fully cover the catalytic pocket of IDO1 was set up with a dimension of 30 × 28 × 30 Å. Molecular drawings were generated using PyMOL (ver. 2.3.4, Schrödinger LLC, New York, NY, USA).

## 4. Conclusions

Here, to adequately analyze the hIDO1 activity, an electrochemical strategy was applied. The nanoporous gold electrode enabled the electrochemically driven hIDO1 reaction to take place, and the enzymatic activity could be measured without concern derived from the reduction reagents used in the conventional method. By using the present system, we performed screening for hIDO1 inhibitors and successfully discovered a strong inhibitor for hIDO1. These results indicate the efficacy of the proposed electroanalytical method in the development of drug discovery for kynureine pathway-targeted immunotherapy.

## Data Availability

Data generated and/or analyzed during this study are within the article or available from the corresponding authors upon reasonable request.

## Supplementary Materials

The following supporting information can be downloaded at https://www.mdpi.com/article/10.3390/ph18030352/s1: Table S1: Docking results of potent inhibitor MTS-7 against IDO1. The top 10 docking modes (docking poses) with their binding energies and the r.m.s.d. values (Å) relative to the best docking mode are presented.
