# Supplementary material for: Electrochemical Analysis and Inhibition Assay of Immune-Modulating Enzyme, Indoleamine 2,3-Dioxygenase
Source: Pharmaceuticals (Basel). 2025 Feb 28;18(3):352. doi: 10.3390/ph18030352 (PMC11944389; doi:10.3390/ph18030352)
Supplement: Supplementary file 1 [file pharmaceuticals-18-00352-s001.zip › pharmaceuticals-3475997-supplementary.pdf]

– Supplementary Table –

Electrochemical Analysis and Inhibition Assay of Immune-Modulating Enzyme, Indoleamine 2,3-Dioxygenase

Yasuhiro Mie *, Chitose Mikami, Yoshiaki Yasutake, Yuki Shigemura, Taku Yamashita and Hirofumi Tsujino

** Corresponding Author: Yasuhiro Mie (yasuhiro.mie@aist.go.jp)*

Supplementary Table S1. Docking results of potent inhibitor MTS-7 against IDO1. The top 10 docking modes (docking poses) with their binding energies and the r.m.s.d. values (Å) relative to the best docking mode are presented.

Mode	Affinity (kcal/mol)	Distances from the best mode (Å)	
		r.m.s.d. lower bound[1]	r.m.s.d. upper bound[2]
1	-8.30	0	0
2	-8.14	1.45	1.89
3	-8.11	0.29	1.30
4	-8.10	2.99	9.06
5	-8.06	1.73	2.30
6	-8.03	1.46	2.14
7	-8.02	3.00	9.12
8	-7.99	0.98	1.16
9	-7.96	1.45	2.02
10	-7.95	1.12	1.68

[1]R.m.s.d. lower bound represents the root mean square deviation of the distances between each atom in one conformation and the closest atom of the same element type in the other conformation (best mode). [2]R.m.s.d upper bound represents the root mean square deviation of the distances between each atom in one conformation and the corresponding atom in the other conformation (best mode), ignoring any symmetry. The best docking mode is shown in Fig. 8.
